# ﻿Species discrimination of novel chloroplast DNA barcodes and their application for identification of *Panax* (Aralioideae, Araliaceae)

**DOI:** 10.3897/phytokeys.188.75937

**Published:** 2022-01-06

**Authors:** Nguyen Nhat Linh, Pham Le Bich Hang, Huynh Thi Thu Hue, Nguyen Hai Ha, Ha Hong Hanh, Nguyen Dang Ton, Le Thi Thu Hien

**Affiliations:** 1 Institute of Genome Research, Vietnam Academy of Science and Technology, 18 Hoang Quoc Viet, Cau Giay, Hanoi, Vietnam Institute of Genome Research, Vietnam Academy of Science and Technology Hanoi Vietnam; 2 Graduate University of Science and Technology, Vietnam Academy of Science and Technology, 18 Hoang Quoc Viet, Cau Giay, Hanoi, Vietnam Graduate University of Science and Technology Hanoi Vietnam

**Keywords:** DNA barcode, *Panax* genus, *Panaxvietnamensis*, *petB*, *trnE-trnT*, *trnQ-rps16*, *trnS-trnG*

## Abstract

Certain species within the genus *Panax* L. (Araliaceae) contain pharmacological precious ginsenosides, also known as ginseng saponins. Species containing these compounds are of high commercial value and are thus of particular urgency for conservation. However, within this genus, identifying the particular species that contain these compounds by morphological means is challenging. DNA barcoding is one method that is considered promising for species level identification. However, in an evolutionarily complex genus such as *Panax*, commonly used DNA barcodes such as nrITS, *matK*, *psbA-trnH*, *rbcL* do not provide species-level resolution. A recent *in silico* study proposed a set of novel chloroplast markers, *trnQ-rps16*, *trnS-trnG*, *petB*, and *trnE-trnT* for species level identification within *Panax*. In the current study, the discriminatory efficiency of these molecular markers is assessed and validated using 91 reference barcoding sequences and 38 complete chloroplast genomes for seven species, one unidentified species and one sub-species of *Panax*, and two outgroup species of *Aralia* L. along with empirical data of *Panax* taxa present in Vietnam via both distance-based and tree-based methods. The obtained results show that *trnQ-rps16* can classify with species level resolution every clade tested here, including the highly valuable *Panaxvietnamensis* Ha et Grushv. We thus propose that this molecular marker to be used for identification of the species within *Panax* to support both its conservation and commercial trade.

## ﻿Introduction

The genus *Panax* L. is well-known in culinary and medicinal traditions in many countries including China, Korea, Japan, and Vietnam. Its species produce ginsenosides, also known as ginseng saponins with strong antioxidant, antidiabetic, antitumor, and neuroprotective activities (Jung et al. 2017; Patel and Rauf 2017). Due to their high commercial demand, many species within *Panax* have been over-harvested and are at risk of extinction (Case et al. 2007; McGraw et al. 2013; Manzanilla et al. 2018). In Vietnam, there are three species of *Panax* including *Panaxvietnamensis* Ha et Grushv., *Panaxstipuleanatus* H.T.Tsai et K.M.Feng, and *Panaxbipinnatifidus* Seem., all recorded and classified as endangered. Of these species *P.vietnamensis* is endemic to Vietnam and is considered to have the highest medical potential and is therefore the most commercially valuable (Nguyen 2005; Nguyen et al. 2007). Two varieties of *P.vietnamensis*, Panaxvietnamensisvar.fuscidiscus K.Komatsu, S.Zhu et S.Q.Cai and Panaxvietnamensisvar.langbianensis N.V.Duy. V.T.Tran et L.N.Trieu, are also present in Vietnam (Phan et al. 2013; Nong et al. 2016). Recently, an unidentified sample of *Panax* discovered on Puxailaileng Mountain of Nghe An Province was also reported by Phan et al. (2014) and referred hereafter as “*Panax* sp. Puxailaileng”.

Historically, morphological methods have been used to identify ginseng species, though this is challenging due to how similar different ginseng species can appear. Incorrect identification can lead to unintentional or intentional mislabeling and adulteration with low-quality ginsengs, and ultimately affect the consumers’ health and damage the providers’ integrity. Recently, molecular methods have been shown to be efficient for solving problems related to species identification. However, the most commonly used barcoding sequences are challenging to use in the genus *Panax*, because these often lack sufficient variability to unambiguously identify the species (Komatsu et al. 2001; Janzen et al. 2009; Hollingsworth et al. 2011; Zuo et al. 2011; Li et al. 2015). According to Zuo et al. (2011)*rpoC1*, *rbcL*, and *rpoB* were the low discriminatory with only four to eight variable sites. The region *psbK-psbI* had the higher discriminatory ability but low chance of successful sequencing (Janzen et al. 2009). Furthermore, *psbA*-*trnH* sequence analysis was reported inaccurate because of the complicated microevolution (Li et al. 2015). The attempts using above loci along with *matK*, *trnD* and *ycf1* for identifying species in the genus *Panax* also were unable to completely solve the challenge (Komatsu et al. 2001; Shi et al. 2015). Other newly proposed InDel (Nguyen et al. 2017) and dCAPS markers (Nguyen et al. 2020) were also developed but the requirement for multi-locus analyses is time-consuming and labor-intensive. This raises the need to develop better molecular markers for identification of the species within *Panax* (Shneyer 2009; Li et al. 2015; Manzanilla et al. 2018).

Previously performed an *in silico* analysis indicated that the chloroplast DNA markers *trnQ-rps16*, *trnE-trnT*, *petB*, and *trnS-trnG* had high species identification potential within the genus *Panax* (Manzanilla et al. 2018) and could be used in routine classification processes. In the present study, we assess and experimentally test the discriminatory efficiency of these commonly used and novel chloroplast markers in classifying species of *Panax* with an emphasis on those distributed in Vietnam.

## ﻿Materials and methods

### ﻿Plant materials

**Figure 1.**
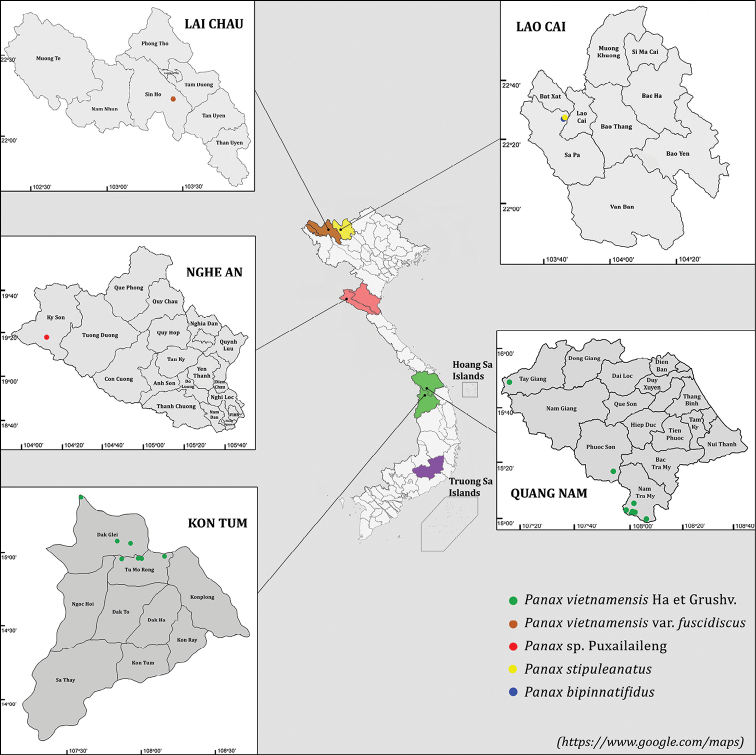
Distribution of *Panax* in Vietnam and sample locations. *P.vietnamensis* (green) collected in Quang Nam and Kon Tum Provinces. P.vietnamensisvar.fuscidiscus (brown) collected in Lai Chau Province. *Panax* sp. Puxailaileng (pink) collected in Nghe An Province. *P.bipinnatifidus* (blue) and *P.stipuleanatus* (yellow) collected in Lao Cai Province. The natural distribution of *P.vietnamensis*, P.vietnamensisvar.fuscidiscus, and *Panax* sp. are marked as green, brown, and pink, respectively. The wild habitat for *P.bipinnatifidus* and *P.stipuleanatus* is shown in yellow, and the purple area represents the distribution region of P.vietnamensisvar.langbiangensis (not included in this study).

Leaf samples of five taxa belonging to the genus *Panax* were collected in the North and Central Vietnam (Table 1, Fig. 1). These included twenty-two samples of *P.vietnamensis* from eighteen distinct populations distributed on Ngoc Linh Mountain in Quang Nam and Kon Tum Provinces, samples of P.vietnamensisvar.fuscidiscus and *Panax* sp. Puxailaileng collected in their natural habitats from Lai Chau and Nghe An Provinces, respectively, as well as, *P.stipuleanatus* and *P.bipinnatifidus* gathered from Lao Cai Province. All specimens were morphologically identified by plant taxonomists Nguyen Tap and Nguyen Quoc Binh using identification keys (Ha and Grushvitzky 1985; Nguyen 2005; Phan et al. 2013; Tran et al. 2016) and deposited at the Vietnam National Museum of Nature (VNMN). P.vietnamensisvar.langbianensis is narrowly distributed in Lang Bian Mountain of Southern Vietnam and we were not able to collect samples from it. All lab and bioinformatics work was conducted at the Institute of Genome Research, Vietnam Academy of Science and Technology.

**Table 1.** Sample collection information.

Sample ID	Collector	Collection date	Collected location		
			Coordinates	District	Province
***P.vietnamensis***					
TL25	Luong Duc Toan	10/16/2017	15°01.17'N, 108°00.76'E	Nam Tra My	Quang Nam
CP13	Luong Duc Toan	10/16/2017	15°01.40'N, 108°03.10'E	Nam Tra My	Quang Nam
TN22	Luong Duc Toan	10/16/2017	15°00.94'N, 108°03.08'E	Nam Tra My	Quang Nam
D42	Le Thi Thu Hien	09/28/2018	15°00.94'N, 108°02.58'E	Nam Tra My	Quang Nam
D43	Le Thi Thu Hien	09/28/2018	15°00.94'N, 108°02.58'E	Nam Tra My	Quang Nam
D11	Le Thi Thu Hien	09/28/2018	15°00.94'N, 108°02.58'E	Nam Tra My	Quang Nam
D6	Le Thi Thu Hien	09/28/2018	15°00.94'N, 108°02.58'E	Nam Tra My	Quang Nam
Q1	Le Thi Thu Hien	09/28/2018	15°02.53'N, 108°02.72'E	Nam Tra My	Quang Nam
B42	Le Thi Thu Hien	09/28/2018	15°03.11'N, 107°97.97'E	Nam Tra My	Quang Nam
ML043	Luong Duc Toan	10/11/2017	15°03.20'N, 107°97.90'E	Nam Tra My	Quang Nam
TL27	Luong Duc Toan	10/11/2017	15°03.18'N, 107°97.91'E	Nam Tra My	Quang Nam
TT15	Luong Duc Toan	10/11/2017	14°96.41'N, 108°10.05'E	Nam Tra My	Quang Nam
TR2	Luong Duc Toan	10/11/2017	15°07.73'N, 108°00.76'E	Nam Tra My	Quang Nam
PL073	Luong Duc Toan	10/11/2017	15°27.50'N, 107°87.90'E	Phuoc Son	Quang Nam
TG07	Luong Duc Toan	10/11/2017	15°79.20'N, 107°25.90'E	Tay Giang	Quang Nam
NLay1	Le Thi My Hao	10/11/2017	14°59.60'N, 108°14.80'E	Tu Mo Rong	Kon Tum
MR3	Le Thi My Hao	10/11/2017	14°97.08'N, 107°99.90'E	Tu Mo Rong	Kon Tum
TX1	Le Thi My Hao	10/11/2017	14°96.10'N, 107°95.40'E	Tu Mo Rong	Kon Tum
MR7	Le Thi My Hao	10/11/2017	14°97.10'N, 107°89.50'E	Tu Mo Rong	Kon Tum
NL1	Le Thi My Hao	10/11/2017	15°06.20'N, 107°94.40'E	Dak Glei	Kon Tum
X1	Le Thi My Hao	10/11/2017	15°07.60'N, 107°83.20'E	Dak Glei	Kon Tum
MH1	Le Thi My Hao	10/11/2017	15°73.00'N, 107°54.43'E	Dak Glei	Kon Tum
**P.vietnamensisvar.fuscidiscus**					
SLC	Nguyen Tien Dung	07/31/2015	22°20.00'N, 103°42.40'E	Sin Ho	Lai Chau
***Panax* sp. Puxailaileng**					
SNA	Nguyen Tien Dung	12/07/2015	19°53.06'N, 104°33.89'E	Ky Son	Nghe An
***P.stipuleanatus***					
TTH	Nguyen Tien Dung	08/26/2015	22°40.86'N, 103°80.67'E	Sa Pa	Lao Cai
***P.bipinnatifidus***					
SVD	Nguyen Tien Dung	08/26/2015	22°40.86'N, 103°80.67'E	Sa Pa	Lao Cai

### ﻿DNA extraction, amplification, and sequencing of previously used and novel markers

Total genomic DNA was extracted from leaf specimens using GeneJET Plant Genomic DNA Purification Kit (Thermo Fisher Scientific, USA) with the provided protocol. The concentration of genomic DNA was determined using a NanoDrop Spectrophotometer 2000 (Thermo Fisher Scientific, USA). Primer pairs for amplification of *psbA*-*trnH*, *matK* and *rbcL* regions were designed based on available sequences deposited in GenBank, and for ITS region primers were designed as previously reported (Sun et al. 1994). Primers for the four barcodes identified *in silico* were designed based on the chloroplast genome sequence of *P.vietnamensis* (Manzanilla et al. 2018) and are shown in Table 2. Each target DNA region was amplified in a PCR volume of 20 μL containing 1X DreamTaq buffer, 200 mM of each dNTP, 2.5 μM of each primer, 1.5 mM MgCl_2_, 0.75 units of Dream Taq DNA polymerase (Thermo Fisher Scientific, USA) and 50 ng of template DNA. PCR amplification was performed on a Mastercycler (Eppendorf, Germany) using the following conditions: 2 min at 94 °C initial denaturation, 35 amplification cycles (30 s at 94 °C denaturation, 20 s at 55–62 °C annealing, and 1 min at 72 °C extension), 5 min at 72 °C extension, and a final hold at 4 °C. The amplified products were purified using GeneJET PCR Purification Kit (Thermo Fisher Scientific, USA) as described by the manufacturer, then screened on a 1% agarose gel. Purified products were sequenced using ABI 3500 Genetic Analyzer applying BigDye Terminator v3.1 Cycle Sequencing Kit (Thermo Fisher Scientific, USA).

**Table 2.** Primers used in this study.

Region	Primer name	Sequence (5’-3’)	Approximate amplicon length (bp)
ITS	ITS_AB_101	ACGAATTCATGGTCCGGTGAAGTGTTCG	650
ITS	ITS_AB_102	TAGAATTCCCCGGTTCGCTCGCCGTTAC	650
*matK*	MatK_F1A	ACYGTATTTTATGTTTACGACG	750
*matK*	MatK_R1A	TCCATHTDGAAATCTTGGTTCA	750
*psbA-trnH*	PsbA_trnH_PF	ACCCGGTCTTAGTGTATACGAG	390
*psbA-trnH*	PsbA_trnH_PR	TTCACTGCCTTGATCCACTTGG	390
*rbcL*	RbcL_PF	AGTGTTGGATTCAAGCTGGTG	550
*rbcL*	RbcL_PR	TGGTTGTGAGTTCACGTTCT	550
*trnQ-rps16* (1)	Pv_trnQ_rps16_F	GAAGATTTAGGTCCTTAGTCGTTCG	590
*trnQ-rps16* (1)	Pv_trnQ_rps16_R	GATTCAGCATTCCCAGAGAATTGG	590
*trnS-trnG* (2)	Pv_trnS_trnG_F	GCCGCTTTAGTCCACTCAGC	660
*trnS-trnG* (2)	Pv_trnS_trnG_F	GTGTTGACATTTTTCGTGGGGG	660
*petB* (3)	Pv_petB_F	AATATTCAGACCTCGCGGCC	580
*petB* (3)	Pv_petB_R	GGCTCAAGCAAAACACCCAA	580
*trnE-trnT* (4)	Pv_trnE_trnT_F	GAGTGGTTGGTCCGTCAGAA	520
*trnE-trnT* (4)	Pv_trnE_trnT_R	CATGGCGTTACTCTACCGCT	520

### ﻿Nucleotide matrix construction

Raw sequencing data were checked for quality and cleaned using BioEdit version 7.0.9 (Hall 1999). Ambiguous nucleotides and poor signal regions were removed to avoid incorrect alignment in further analyses. A nucleotide matrix was assembled for both individual and concatenated markers. A matrix from the newly obtained and 91 reference barcoding sequences, and 38 complete chloroplast genomes representing seven species of *Panax* (*P.vietnamensis*, *P.stipuleanatus*, *P.bipinnatifidus*, *Panaxginseng* C.A.Mey, *Panaxjaponicus* (T.Nees) C.A.Mey, *Panaxnotoginseng* (Burkill) F.H.Chen ex C.Y.Wu et K.M.Feng, *Panaxquinquefolius* L.), one unidentified species of *Panax* (*Panax* sp. Puxailaileng), and one sub-species of *Panax* (P.vietnamensisvar.fuscidiscus), and two species of *Aralia* L. (*Araliaelata* (Miq.) Seem. and *Araliaundulata* Hand.-Mazz. in Broterus) used as outgroup (Suppl. material 1) were globally aligned using MAFFT version 7.407 (Katoh et al. 2002) followed by local re-alignment with MUSCLE version 3.8.1551 (Edgar 2004). Manual adjustments were made when necessary to improve the matrix. Variable sites, Parsimony informative (PI) sites, mean pairwise distances, and intra/interspecific mean distances were calculated based on nucleotide matrix by MEGAX software (Kumar et al. 2018).

### ﻿Genetic distance-based methods for species discrimination

Pairwise summary and pairwise explorer modules in TaxonDNA version 1.8 (Meier et al. 2006) were used to calculate the distribution of intra/interspecific pairwise distances for barcoding gap analysis in order to analyze the space between intra- and interspecific distances using the Kimura-2-parameter (K2P) nucleotide substitution model. The Best Match/ Best Close Match (BM/ BCM) modules in TaxonDNA were also used to assess species discrimination power of the analyzed markers using the K2P distance as a model. Thresholds for the best close match were computed from the pairwise summary.

### ﻿Tree-based method for species discrimination

The best substitution model for each matrix was searched for using the jModelTest2 (Darriba et al. 2012). Phylogenetic trees based on Maximum Likelihood (ML) method were constructed by both RAxML version 8.2.10 (Stamatakis 2014) and IQTREE version 1.6.12 (Bui et al. 2020). Two species *A.undulata* and *A.elata*, from the sister genus *Aralia* were used as outgroups. ML tree searches were performed with bootstrap calculation at 1000 bootstrap replicates. ML trees were then used to perform species delimitation using mPTP version 0.2.4 (Kapli et al. 2017) with two Markov chain Monte Carlo (MCMC) runs, one million steps for each run, and Likelihood ratio test set to 0.01.

## ﻿Results

### ﻿Amplification and sequencing efficiency

To evaluate the species discrimination efficiency for both the commonly used as well as newly proposed DNA markers for *Panax* we assessed the amplification success as well as the amplicon lengths. Bidirectional Sanger DNA sequencing of each fragment showed the amplicon lengths to be as follows: ITS 618–619 bp, *matK* 751 bp, *psbA-trnH* 352–361 bp, *rbcL* 521 bp, *trnQ-rps16* 575–590 bp, *trnS-trnG* 648–658 bp, *petB* 576–577 bp, and *trnE-trnT* 490–514 bp. ITS and *matK* did not amplify efficiently despite optimization of PCR amplification conditions, while other chloroplast regions were easily amplified. Despite some challenges, both PCR amplification and sequencing were successful for all regions (Table 3).

**Table 3.** Amplification and sequence information for all analyzed markers and their combinations.

**Marker**	**Amplification/ Sequencing success rate (%)**	**Matrix size (bp)**	**Variable sites (%)**	**No. of PI sites**	**Mean pairwise distance**	**Intraspecific distances (mean)**	**Interspecific distances (mean)**
**ITS**	100/ 100	623	17.17	75	0.0259	0.0000 –0.0292 (0.0107)	0.0082 –0.0400 (0.0261)
***matK***	100/ 100	751	4.26	29	0.0054	0.0000- –0.0016 (0.0003)	0.0000 –0.0216 (0.0091)
***psbA-trnH***	100/ 100	362	10.22	27	0.0175	0.0000 –0.0029 (0.0010)	0.0000 –0.0297 (0.0212)
***rbcL***	100/ 100	521	2.50	11	0.0061	0.0000 –0.0007 (0.0002)*	**0.0019 –0.0101 (0.00615)**
***trnQ-rps16* (1)**	100/ 100	657	6.54	35	0.0116	**0.0000 –0.0025 (0.0007)**	**0.0067 –0.0222 (0.0131)**
***trnS-trnG* (2)**	100/ 100	674	5.34	22	0.0068	0.0000 –0.0027 (0.0005)	0.0017 –0.0133 (0.0082)
***petB* (3)**	100/ 100	591	5.58	30	0.0164	0.0000 –0.0025 (0.0004)	0.0013 –0.0340 (0.0196)
***trnE-trnT* (4)**	100/ 100	614	13.84	16	0.0075	**0.0000 –0.0004 (0.0001)**	**0.0039 –0.0274 (0.0108)**
**1+2**	100/ 100	1331	5.94	57	0.0090	**0.0000- –0.0021 (0.0006)**	**0.0047 –0.0167 (0.0105)**
**1+3**	100/ 100	1248	6.09	65	0.0139	**0.0000- –0.0025 (0.0006)**	**0.0040 –0.0251 (0.0164)**
**1+4**	100/ 100	1271	10.07	51	0.0096	**0.0000 –0.0014 (0.0004)**	**0.0054 –0.0238 (0.0120)**
**2+3**	100/ 100	1265	5.45	52	0.0112	**0.0000 –0.0014 (0.0005)**	**0.0017 –0.0210 (0.0135)**
**2+4**	100/ 100	1288	9.39	38	0.0071	**0.0000 –0.0017 (0.0003)**	**0.0034 –0.0195 (0.0093)**
**3+4**	100/ 100	1205	9.79	46	0.0121	**0.0000 –0.0013 (0.0003)**	**0.0025 –0.0240 (0.0154)**
**1+2+3**	100/ 100	1922	5.83	87	0.0113	**0.0000 –0.0016 (0.0005)**	**0.0036 –0.0196 (0.0134)**
**1+2+4**	100/ 100	1945	8.43	73	0.0086	**0.0000 –0.0016 (0.0005)**	**0.0045 –0.0199 (0.0106)**
**1+3+4**	100/ 100	1862	8.65	81	0.0119	**0.0000 –0.0017 (0.0004)**	**0.0040 –0.0213 (0.0146)**
**2+3+4**	100/ 100	1879	8.20	68	0.0101	**0.0000 –0.0011 (0.0004)**	**0.0027 –0.0186 (0.0127)**
**1+2+3+4**	100/ 100	2536	7.77	103	0.0104	**0.0000 –0.001 (0.0005)**	**0.0037 –0.0181 (0.0128)**

*Markers with the barcoding gap were displayed in bold format. Grey = common barcoding markers, Green = novel barcoding markers; Yellow = combinations of two novel markers; Orange = combinations of three novel markers; Dark grey = combination of all four novel markers.

### ﻿Nucleotide matrix

The nucleotide matrices for the amplified markers and complemented with the 89 reference barcoding sequences and 36 complete chloroplast genomes from the seven species of *Panax*, one unidentified species and one sub-species of *Panax* present in GenBank, showed that the matrix sizes ranged from 362 to 751 bp for individual markers and 1205 to 2536 bp for concatenated markers (Table 3). Among all individual and concatenated markers, ITS possessed the highest proportion of variable sites (17.17%), followed by *trnE-trnT* (13.84%), *psbA-trnH* (10.22%), and *trnQ-rps16* and *trnE-trnT* combined (10.07%). ITS also had the most divergence (0.0259) when calculating the mean pairwise distances for each barcode, followed by *psbA-trnH* (0.0175) and *petB* (0.0164) (Table 3). Higher numbers of variable sites and pairwise distances indicate higher species divergence, though a previous study has suggested that the proportion of variable sites may not affect a marker’s classification ability (Manzanilla et al. 2018).

### ﻿Species discrimination power assessment for different markers

#### Genetic distance-based and sequence similarity-based analyses

**Figure 2.**
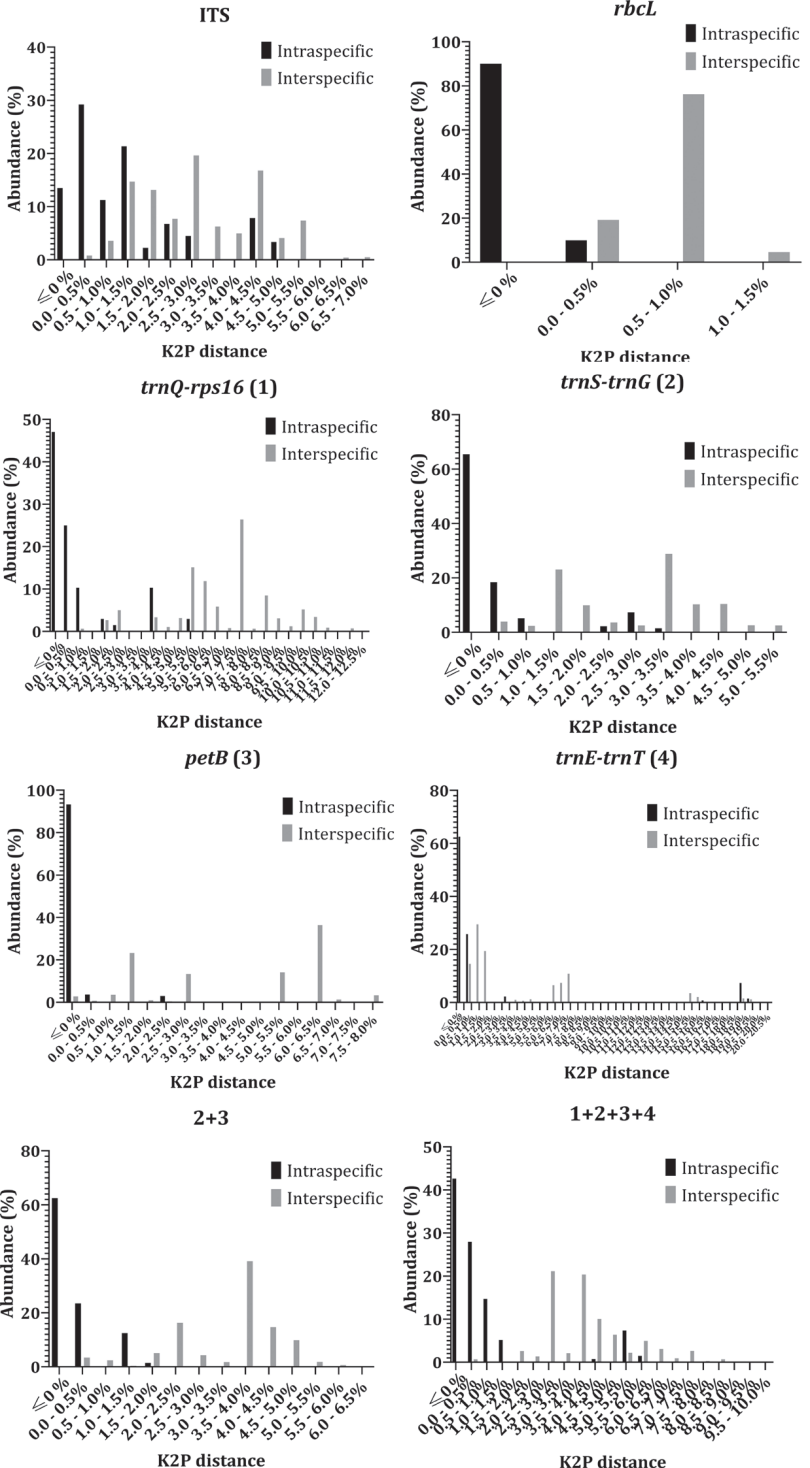
Percent relative abundance in distribution of intra/interspecific K2P pairwise distances estimated for markers.

Distance-based classification methods rely on intraspecific and interspecific distances to set a threshold to distinguish distinct species. In this study, genetic distances were calculated between individuals both within and between species using MEGAX and Pairwise Explorer (TaxonDNA). Due to the complexity in the species group consisting of *P.bipinnatifidus* and *P.stipuleanatus*, these two species were treated as a single group when calculating pairwise distances and assessing the species classification ability of different markers. For interspecific distances, MEGAX computed the average distance of all pairwise distances between each two species while TaxonDNA returned all the distances for every pair of sequences. According to the distances obtained from MEGAX a barcoding gap exists in *rbcL*, *trnQ-rps16*, *trnE-trnT*, and all combined markers (Table 3). However, distribution analysis of pairwise distances in TaxonDNA shows that there was no barcoding gap in eight individual and eleven concatenated marker pairs (Fig. 2). The overlap of intraspecific and interspecific distance distribution was mainly due to the complex genetic distances inside *P.ginseng* species and similarity between them and *P.quinquefolius*. High divergence within species and low diversity between species in a complex genus like *Panax* results in difficulties in setting threshold values for species discrimination using distance-based methods.

**Figure 3.**
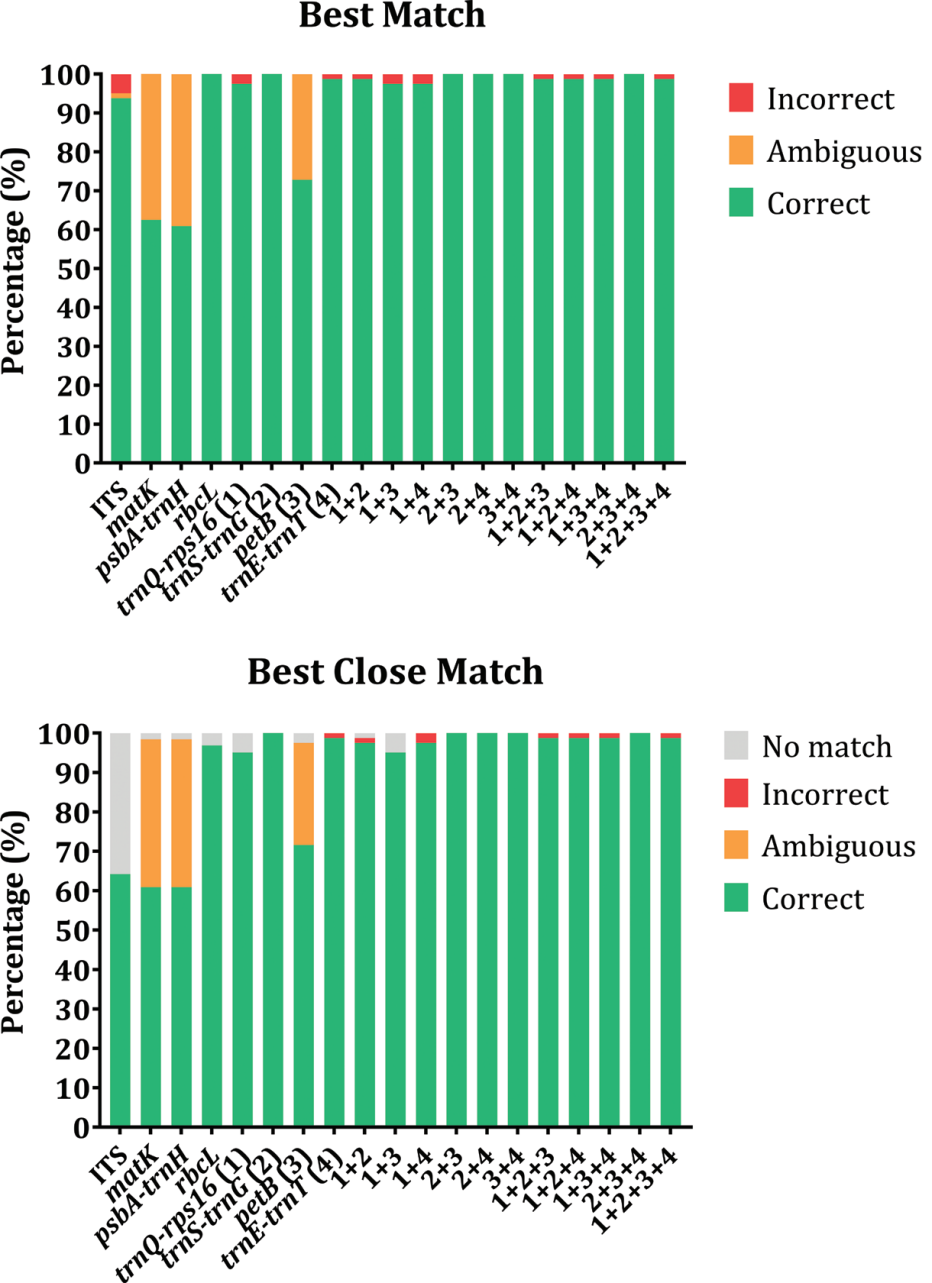
Successful identification rates among analyzed barcodes by Best Match and Best Close Match function.

BM/BCM analysis from TaxonDNA discriminates species based on similarity between sequences. For separated barcodes, analysis results showed that *trnS-trnG* and *rbcL* regions had the strongest discriminatory power with 100% correct identification for both BM calculations, followed by *trnE-trnT* (98.76%), *trnQ-rps16* (97.53%), and ITS (93.82%). BCM analysis returned more stringent calculations of successful identified sequences than BM with 100% for *trnS-trnG*, 98.76% for *trnE-trnT*, 96.87% for *rbcL*, and 95.06% for *trnQ-rps16*. Markers that had the lowest identification success rate were *petB* (BM: 72.83%, BCM: 71.60%), *matK* (BM: 62.50%, BCM: 60.93%), and *psbA-trnH* (BM: 60.93%, BCM: 60.93%). Combinations made from the four newly proposed markers were also estimated for species identification tests. Discriminatory abilities of concatenated markers were observed to be slightly better than most separated barcodes. Combinations 2+3, 2+4, 3+4, and 2+3+4 showed correct classification rates of 100% for both BM and BCM calculations (Fig. 3).

#### Tree-based analysis

**Figure 4.**
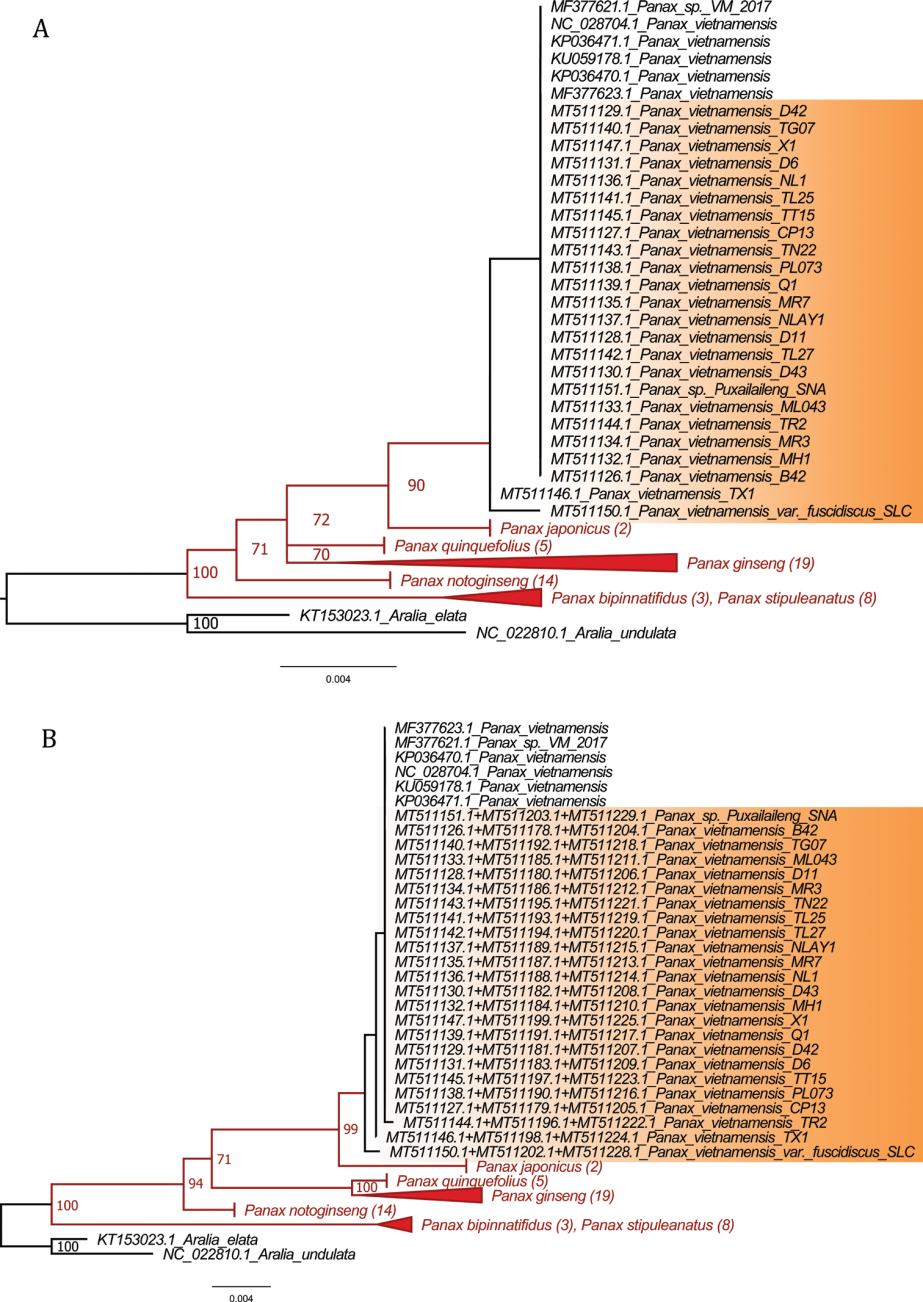
Results of mPTP species delimitation analysis for several markers based on ML trees **A** Species delimitation for marker *trnQ-rps16***B** Species delimitation for the combination of markers 1+3+4. Bootstrap values are displayed on the branches. The red branches represent supported species delimitations. Sequences highlighted in orange originate from this study.

**Figure 5.**
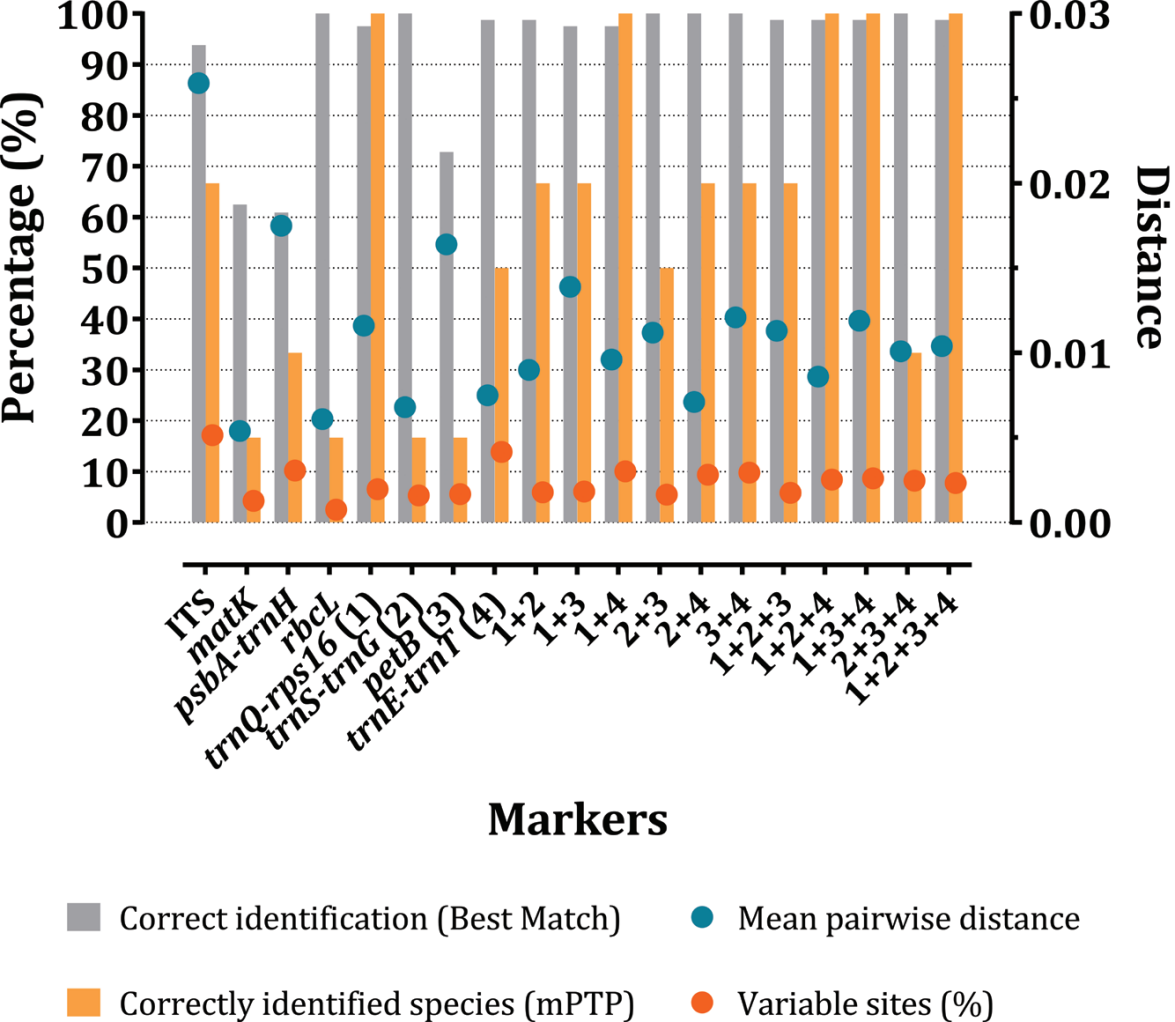
Percentage of variable sites, mean pairwise distances, and correct classification percentages of all markers and combinations

Both separate and concatenated matrices were used to reconstruct ML trees. We found that most of the markers could separate most of the clades with strong bootstrap support, with the exception of *P.bipinnatifidus* and *P.stipuleanatus*. These sister species had poor branch structure and weak support values. The taxonomic circumscription of *P.bipinnatifidus* has been controversial. Recent studies from Nong et al. (2016), Pham et al. (2020) suggested identifying the leaflet ginseng, which was previously recorded as *P.bipinnatifidus* in Vietnam, as *P.stipuleanatus*. Based on morphological characteristics and ITS region, Wen and Zimmer (1996) suggested that the division of the leaflet does not warrant recognition of a novel species or variety. We therefore grouped these species into one group since the initial analysis steps and clade complexity excluded further classification tests. High intraspecific divergence in *P.ginseng* resulted in this clade being divided into two subgroups in the phylogenetic analysis. In contrast, although there are nucleotide differences between sequences of *P.vietnamensis* TX1, P.vietnamensisvar.fuscidiscus SLC, and other samples of *P.vietnamensis*, our analysis clearly showed that all samples of *P.vietnamensis*, and a taxon *Panax* sp. Puxailaileng belonged to the same clade with strong support (Fig. 4). Phylogenetic trees were also used to estimate the species delimitation using mPTP. The results indicated that among eight individual markers only *trnQ*-*rps16* region could classify all six clades of *Panax* with strong support. Other regions performed more poorly in delimitation of tested taxa and only weakly supported speciation between *P.ginseng* and *P.quinquefolius* and/or *P.vietnamensis* and *P.japonicus*. The ITS region is an exception as its nucleotide sequence has the highest variability among species leading to over-splitting of sequences into many small subgroups. The excessive segregation limited the discriminatory ability of this ITS marker at species level. For concatenated barcodes, 1+4, 1+2+4, 1+3+4, and 1+2+3+4 showed the ability to classify all six clades in the genus *Panax* (Figs 4, 5).

#### Selecting markers for identification of *Panax* spp.

Incongruence between genetic distance-based, sequence similarity-based and tree-based methods has led to difficulties in choosing robust markers for species discrimination in complex genera like *Panax*. Here we examined the identification abilities of two methods for four newly proposed markers and combinations thereof in comparison with four commonly used barcodes (Fig. 5). Distance-based methods failed to detect the barcoding gap between intraspecific and interspecific distances for analyzed markers due to the complex divergence in sister species *P.ginseng* and *P.quinquefolius*. *RbcL* had the least overlapping intra/interspecific distances, but also had the lowest variation in pairwise distance. This leads to low resolution in species classification. Barcoding analysis based on sequence similarity showed high correct identification percentages for two of the four common barcodes and three of the four novel barcodes. All concatenated markers showed high identification power, but this was not the case for classification results calculated by tree-based methods. The highest identification rates were observed in *trnQ-rps16*, 1+4, 1+2+4, 1+3+4, and 1+2+3+4 regions with mPTP analysis. Other robust markers identified by TaxonDNA had lower species resolution in mPTP. We thus propose that *trnQ-rps16* is the best single marker for species identification in the *Panax* genus since it provides the best classification resolution in both sequence similarity-based and tree-based analyses.

## ﻿Discussion

Phylogenetic studies on *Panax* using different DNA barcodes, different reference sequences or samples have resulted in conflicting tree topologies and clade placements for several species (Wen and Zimmer 1996; Komatsu et al. 2001; Zuo et al. 2011; Ali et al. 2012; Le et al. 2017; Manzanilla et al. 2018). For example, two sister species *P.bipinnatifidus* and *P.stipuleanatus* were separated into two distant clades based on the combined dataset of six markers by Zuo et al. (2011). Meanwhile, these two above taxa could not be separated into two clades using the ITS region alone (Wen and Zimmer 1996). This inability to resolve a clear sister relationship between these two species was also supported by the four novel markers assessed in our study. Similarly, *P.vietnamensis* was reported to belong to the same clade as Panaxjaponicusvar.major (Burkill) C.Y.Wu et Feng, Panaxpseudoginsengsubsp.himalaicus H.Hara based on 18S rRNA and *matK* (Komatsu et al. 2001), and to be closely related to *Panaxzingiberensis* C.Y.Wu et Feng and *Panaxwangianus* S.C.Sun based on ITS2 (Ali et al. 2012), *P.notoginseng* based on ITS, *matK*, *rbcL*, *psbA-trnH*, and 18S rRNA (Le et al. 2017), as well as *P.japonicus* based on *in silico* data of four potential markers (Manzanilla et al. 2018). With regard to an unidentified sample *Panax* sp. Puxailaileng, our phylogenetic trees based on comprehensive datasets of the marker *trnQ-rps16* or combined markers 1+3+4 (Fig. 4) obviously revealed this taxon belonged to the same clade with all samples of *P.vietnamensis*. Relevant bootstrap values at 90–99 by Maximum Likelihood method indicated that confidence intervals were eligible for genetic correlation of these samples. The obtained results on these novel markers are congruent with or different from previous studies. *Panax* sp. Puxailaileng was suggested to be *P.vietnamensis* based on its morphological characteristics and ITS-rDNA sequence, though further studies are still needed to unambiguously resolve its identity (Tran et al. 2016). Similarly, morphology and molecular-based phylogenetic analyses suggested *Panax* sp. Puxailaileng found in the wild in Ky Son District, Nghe An Province were P.vietnamensisvar.fuscidiscus (Pham et al. 2020). In another study, samples of *Panax* sp. collected from Puxailaileng Mountain were reported to be closely related to *P.stipuleanatus* based on the commonly use markers ITS-rDNA and *matK* (Vu et al. 2020). Present results based on empirical data support the results of the *in silico* study by Manzanilla et al. (2018). *P.vietnamensis* is closely related to *P.japonicus*. This apparent contradiction with other studies might result from differences in dataset structures, the number of species and taxa included, and classification methods. Indeed, different approaches can return different results in DNA barcoding analyses (Wen and Zimmer 1996; Komatsu et al. 2001; Zuo et al. 2011; Ali et al. 2012; Le et al. 2017; Manzanilla et al. 2018). The genetic distances calculated in MEGAX suggest that barcoding gaps exist in the markers *rbcL*, *trnQ-rps16*, *trnE-trnT*, and all concatenated markers, whereas TaxonDNA showed overlap between intra and interspecific distances in all analyzed markers (Table 3, Fig. 2). This incongruence could make a tremendous difference in the output of the analyses. Nevertheless, results obtained from the BM/BCM module in TaxonDNA are similar to distances calculated in MEGAX. In general, the discriminatory power assessed in TaxonDNA is higher than in the tree-based method mPTP (Fig. 5). Especially for *rbcL*, distance-based methods provide the highest percentage of correctly identified species while the tree-based method gave the lowest percentage of correctly identified species. The low number of variable sites within some markers can explain the differences in results between the two methods (Fig. 5). Highly conserved regions with low percentages of variable sites might lead to sound results when calculating genetic distances, but might make the construction of a phylogenetic tree challenging. This limitation for distance-based methods can sometimes result in biologically meaningless results (Meier et al. 2008; Ross et al. 2008). However, the comparison between the two methods generated several common similarities for the marker *trnQ-rps16* and a number of different marker combinations with high discriminatory power, e.g., 1+4, 1+2+4, 1+3+4, 1+2+3+4 (Fig. 5).

In the present study, the discriminatory power of four chloroplast markers proposed by Manzanilla et al. (2018), for *Panax* spp. was evaluated. The highly valuable *P.vietnamensis* species and collected samples from eighteen distinct populations across the country were analyzed. The obtained results did not show any significant differences between the populations, which suggests that *trnQ-rps16* marker is appropriate for identification at the species level and does not lead to misidentifications of *P.vietnamensis* regardless of their origins or geographical distributions. Only small differences between experimental and *in silico* analyses were observed. The *in silico* study included only a limited number of *P.vietnamensis* accessions, and this may explain why the empirical data contains more variable sites in *P.vietnamensis*. Our results support the use of *trnQ-rps16* as a single molecular marker for species identification in the genus *Panax*. An advantage of using a single region for species identification is the reduced time and effort necessary for routine analyses, though it can be combined with other markers (*petB*, *trnE-trnT*) when necessary if a single marker is not enough to unambiguously provide a species-level identification. Beside its discriminatory power, *trnQ-rps16* can also be easily amplified.

## Supplementary materials

Supplementary material 1 NCBI accession numbers of DNA barcoding sequences, and complete chloroplast genomes used in this study. Data type NCBI accession numbers of DNA sequences and complete chloroplast genomes Explanation note The NCBI accession numbers of newly obtained and 91 reference barcoding sequences, and 38 complete chloroplast genomes representing seven *Panax* species, one unidentified species and one sub-species of *Panax*, and two outgroup *Aralia* species. https://binary.pensoft.net/file/631059This dataset is made available under the Open Database License (http://opendatacommons.org/licenses/odbl/1.0/). The Open Database License (ODbL) is a license agreement intended to allow users to freely share, modify, and use this Dataset while maintaining this same freedom for others, provided that the original source and author(s) are credited.Nguyen Nhat Linh, Pham Le Bich Hang, Huynh Thi Thu Hue, Nguyen Hai Ha, Ha Hong Hanh, Nguyen Dang Ton, Le Thi Thu Hien
