# Supplementary material for: ﻿Species discrimination of novel chloroplast DNA barcodes and their application for identification of Panax (Aralioideae, Araliaceae)
Source: PhytoKeys. 2022 Jan 6;188:1–18. doi: 10.3897/phytokeys.188.75937 (PMC8758638; doi:10.3897/phytokeys.188.75937)
Supplement: Supplementary material 1 — NCBI accession numbers of DNA barcoding sequences, and complete chloroplast genomes used in this study. [file phytokeys-188-001-s001.pdf]

Supplement File 1. NCBI accession numbers of DNA barcoding sequences, and complete chloroplast genomes used in this study.

Ingroup: ***Panax vietnamensis* Ha et Grushv.**, Voucher B42*, MT511126.1, MT511152.1, MT511178.1, MT511204.1; Voucher CP13*, MT511127.1, MT511153.1, MT511179.1, MT511205.1; Voucher D11*, MT511128.1, MT511154.1, MT511180.1, MT511206.1; Voucher D42*, MT511129.1, MT511155.1, MT511181.1, MT511207.1; Voucher D43*, MT511130.1, MT511156.1, MT511182.1, MT511208.1; Voucher D6*, MT511131.1, MT511157.1, MT511183.1, MT511209.1; Voucher MH1*, MT511132.1, MT511158.1, MT511184.1, MT511210.1; Voucher ML043*, MT511133.1, MT511159.1, MT511185.1, MT511211.1; Voucher MR3*, MT511134.1, MT511160.1, MT511186.1, MT511212.1; Voucher MR7*, MT511135.1, MT511161.1, MT511187.1, MT511213.1; Voucher NL1*, MT511136.1, MT511162.1, MT511188.1, MT511214.1; Voucher NLAY1*, MT511137.1, MT511163.1, MT511189.1, MT511215.1; Voucher PL073*, MT511138.1, MT511164.1, MT511190.1, MT511216.1; Voucher Q1*, MT511139.1, MT511165.1, MT511191.1, MT511217.1; Voucher TG07*, MT511140.1, MT511166.1, MT511192.1, MT511218.1; Voucher TL25*, MT511141.1, MT511167.1, MT511193.1, MT511219.1; Voucher TL27*, MT511142.1, MT511168.1, MT511194.1, MT511220.1; Voucher TN22*, MT511143.1, MT511169.1, MT511195.1, MT511221.1; Voucher TR2*, MT511144.1, MT511170.1, MT511196.1, MT511222.1; Voucher TT15*, MT511145.1, MT511171.1, MT511197.1, MT511223.1; Voucher TX1*, MT511146.1, MT511172.1, MT511198.1, MT511224.1; Voucher X1*, MT511147.1, MT511173.1, MT511199.1, MT511225.1; Voucher VMN-B2148*, MT511239.1, MT511248.1, MT511257.1, MT498787.1; Voucher PV22*, MT511240.1, MT511249.1, MT511258.1, MT498788.1; Voucher PV23*, MT511241.1, MT511250.1, MT511259.1, MT498789.1; Isolate 37, KP036470.1, NC_028704.1; Isolate 38, KP036471.1; Voucher P01_ITS_711_bases, MK979379.1; Voucher P02_ITS_711_bases, MK979380.1; Voucher P04_ITS_698_bases, MK979381.1; Voucher P05_ITS_672_bases, MK979382.1; Voucher P11_ITS_706_bases, MK979387.1; Voucher P13_ITS_706_bases, MK979389.1; Voucher P16_ITS_706_bases, MK979392.1; Isolate ITSLW1, MH345175.1; Isolate ITSLW2, MH345176.1; Isolate ITSLW3, MH345177.1; Voucher Wen_5638_2(F), AY271924.1; Unknown voucher, KU059178.1, MF377623.1. ***Panax vietnamensis* var. *fuscidiscus* K.Komatsu, S.Zhu et S.Q.Cai**, Voucher SLC*, MT511150.1, MT511176.1, MT511202.1, MT511228.1; Isolate ITSJP1, MH345115.1; Isolate ITSJP2, MH345116.1; Isolate ITSJP4, MH345118.1. ***Panax* sp. Puxailaileng**, Voucher SNA*, MT511151.1, MT511177.1, MT511203.1, MT511229.1, MT501804.1; Voucher VM-2017, MF377621.1; Voucher PVM3*, MT501805.1. ***Panax stipuleanatus* H.T.Tsai et K.M.Feng**, Voucher TTH*, MT511149.1, MT511175.1, MT511201.1, MT511227.1 ; Voucher APS58.1*, MT511242.1, MT511251.1, MT511260.1, MT498790.1; Voucher APS58.2*, MT511243.1, MT511252.1, MT511261.1, MT498791.1; Voucher APS58.3*, MT511244.1, MT511253.1, MT511262.1, MT498792.1; Isolate JYH-2016466, MK408920.1; Isolate JYH-2016435, MK408936.1; Isolate JYH-2016437, MK408965.1; Voucher PV01, MK961249.1; Isolate ITSS1, MH345085.1; Isolate ITSS2, MH345086.1; Isolate ITSS7, MH345091.1; Voucher Wen_5631_5(F), AY271922.1; Isolate MG_PPW034 Voucher D1(PE), HQ112441.1; Isolate V_Z155D Voucher Vietnam4(US), HQ112442.1; Unknown voucher,

KX247147.1, NC_030598.1, KY379906.1, MF377622.1. ***Panax bipinnatifidus* Seem.**, Voucher SVD*, MT511148.1, MT511174.1, MT511200.1, MT511226.1; Voucher APB57.1*, MT511245.1, MT511254.1, MT511263.1, MT498793.1; Voucher APB57.2*, MT511246.1, MT511255.1, MT511264.1, MT498794.1; Voucher APB57.3*, MT511247.1, MT511256.1, MT511265.1, MT498795.1; Voucher ITSCY3, MH345137.1; Voucher ITSCY4, MH345138.1; Voucher ITSCY7, MH345141.1; Voucher ITSCY10, MH345144.1; Voucher ITSMX3, MH345167.1; Voucher ITSGS3, MH345181.1; Voucher ITSGS4, MH345182.1; Isolate BY_PPW331 Voucher Z1(PE), HQ112454.1; Isolate H_PPW170 Voucher Wen4942(US), HQ112418.1; Unknown voucher, KX247146.1, MF377620.1. ***Panax ginseng* C.A.Mey**, Isolate Ermaya, KC686332.1; Isolate Gaolishen, KC686333.1; Isolate Damaya, KC686331.1; Cultivar Cheongsun, KM067386.1; Cultivar Gopoong, KM067387.1; Cultivar Gumpoong, KM067388.1; Cultivar Jakyung, KM067389.1; Cultivar Sunpoong, KM067391.1; Cultivar Sunun, KM067392.1; Cultivar Sunhyang, KM067393.1; Cultivar Hwangsook, KM067394.1; Cultivar Chungpoong, KM088019.1; Cultivar YunPoong, KM088020.1; Cultivar Sunone, KM067390.1; Isolate JYH-2016473, MK408938.1; Cultivar R-JLCB, MT125968.1; Cultivar RG-JLCB, MT125969.1; Cultivar Y-JLJA, MT125970.1; Cultivar R-HG, MT125972.1; Cultivar G-JLCB, MT125973.1; Cultivar G-HG-1, MT125974.1; Cultivar G-HG-2, MT125975.1; Cultivar Yunpoong, AF274532.1; Cultivar Chunggyung, AF274534.1; Isolate KGD5, DQ339099.1; Isolate KGD4, DQ339098.1; Cultivar Gopoong; HM446500.1; Cultivar Sunpoong, HM446502.1; Isolate JL_GS002 Voucher Zhou002(PE), HQ112416.1; Cultivar Sunhyang, KF727972.1; Unknown voucher, AY582139.1, NC_006290.1, MH049735.2, KF431956.1. ***Panax japonicus* (T.Nees) C.A.Mey**, Voucher Q079, MH711079.1; Voucher Wen_2459(CS), AY271918.1 ; Voucher PS1477MT03, FJ980424.1 ; Isolate J_PPW071 Voucher J5(PE), HQ112432.1; Voucher GZ130603, KJ559420.1; Voucher GZ130604, KJ559421.1; Isolate ITSZT3, MH345097.1; Isolate ITSZT4, , MH345098.1; Unknown voucher, KP036469.1, NC_028703.1, AF263373.1. ***Panax notoginseng* (Burkill) F.H.Chen ex C.Y.Wu et K.M.Feng**, Isolate LCK-5, MK408925.1; Isolate LCK-1, MK408927.1; Isolate LCK-8, MK408928.1; Isolate LCK-7, MK408931.1, ; Isolate LCK-3, MK408937.1; Isolate LCK-2, MK408945.1; Isolate LCK-4, MK408946.1; Isolate LCK-10, MK408954.1; Isolate LCK-9, MK408955.1; Isolate ITSN2, MH345106.1; Isolate ITSN1, MH345105.1; Isolate ITSN3, MH345107.1; Isolate ITSN5, MH345109.1; Isolate ITSN6, MH345110.1; Isolate ITSLW4, MH345178.1; Voucher Wen_1211(CS), AY271919.1; Isolate WS_PW002 Voucher D7(PE), HQ112434.1; Isolate WS_PW003 Voucher D7(PE), HQ112435.1; Isolate WS_PW007 Voucher D7(PE), HQ112436.1; Unknown voucher, KJ566590.1, NC_026447.1, KP036468.1, KR021381.1, KT001509.1, JX680329.1. ***Panax quinquefolius* L.**, Isolate JYH-2016493, MK408923.1; Isolate JYH-2016494, MK408953.1 ; Cultivar X-JLCB, MT125971.1; Isolate VA_Z164K Voucher Wen6243(US), HQ112440.1; Isolate JL_Z163A Voucher Wen5420(US), HQ112439.1; Voucher T3982, KU886054.1; Unknown voucher, KM088018.1, NC_027456.1, KT028714.1, FJ606755.1.

Outgroup: *Aralia elata* (Miq.) Seem, Isolate ITSA, MH345084.1; Unknown voucher, KT153023.1. ***Aralia undulata* Hand.-Mazz. in Broterus**, Unknown voucher, NC_022810.1, AF273539.1.
